# Preventive Effect of Different Wild Pistachio Oils on Oxidative Stress Markers, Liver Enzymes, and Histopathological Findings in a Metabolic Syndrome Model

**DOI:** 10.22086/gmj.v8i0.1238

**Published:** 2019-01-01

**Authors:** Sanaz Jamshidi, Najmeh Hejazi, Mohammad-Taghi Golmakani, Nader Tanideh, Mina Heidari Esfahani

**Affiliations:** ^1^Department of Clinical Nutrition, School of Nutrition and food sciences, Shiraz University of Medical Sciences, Shiraz, Iran; ^2^Nutrition research center, Department of Clinical Nutrition, School of Nutrition and food sciences, Shiraz University of Medical Sciences, Shiraz, Iran; ^3^Department of Food Science and Technology, School of Agriculture, Shiraz University, Shiraz, Iran; ^4^Stem Cell and Transgenic Research Center, Shiraz University of Medical Sciences, Shiraz, Iran; ^5^Pathology Department, Birjand University of Medical Sciences, Birjand, Iran

**Keywords:** Wild Pistacia, Oils, Liver Function Tests, Insulin Resistance

## Abstract

**Background:**

Wild pistachio (*Pistacia Atlantica mutica*) species with wide distribution in Iran have different nutrition properties and may have therapeutic effects in metabolic syndrome. Metabolic syndrome, as a prevalent health problem, is a main risk factor for different chronic diseases such as diabetes, fatty liver and cardiovascular diseases. The present study aimed to evaluate the preventive effects of mixture, kernel, and hull oil of wild pistachio (WP) on oxidative stress markers, liver function and histopathological findings in metabolic syndrome induced rats.

**Materials and Methods:**

After oil extraction by cold press method and chemical analysis, rats were divided into 6 groups. Group 1 received normal saline; group 2 received 2cc fructose solution and 0.5cc normal saline; and groups 3, 4, 5 and 6 received 2 cc fructose solution and 0.5 cc sunflower oil, mixture, hull and kernel oils of WP for 10 weeks, respectively. Then, glycemic indices, oxidative stress, liver enzymes and histopathological examination were determined using standard laboratory tests.

**Results:**

WP Kernel and mixture oils notably decreased the fasting blood sugar and insulin resistance compared with the fructose group. Insulin level was significantly increased in the kernel oil group (P<0.05). There was no significant difference in oxidative stress, liver enzymes and histopathology parameters among the groups.

**Conclusion:**

Kernel oil of WP improved hyperglycemia, insulin resistance and insulin secretion, but the changes in oxidative stress markers, liver enzymes and histopathologic results were not significant among the groups.

## Introduction

Metabolic syndrome is a combination of metabolic disturbances and is also known as the insulin resistance (IR) syndrome [1, 2]. Atherogenic dyslipidemia, elevated blood pressure, hyperglycemia, and prothrombotic and proinflammatory states are the disorders related to metabolic syndrome, which can increase mortality from cardiovascular diseases [3-5]. IR is an underlying factor of metabolic syndrome, and investigators believe that it can directly cause other metabolic abnormalities [5]. On the other hand, oxidative stress may have destructive effects on individuals with metabolic syndrome [6]. Till date, there are no clear therapeutic approaches for metabolic syndrome; however, it seems that dietary interventions can play an important role in its improvement [7]. Modifiable lifestyle factors such as intake of vegetables and fruits, low-glycemic-index foods, low salt, antioxidants, and dietary fat intake and its quality can improve the metabolic profile [6, 8, 9]. Total fat intake is associated with IR and risk of metabolic syndrome. As the replacement of saturated fatty acids with monounsaturated fatty acids (MUFA) can improve the IR and risk of metabolic syndrome, introducing local sources of MUFA may have preventive effects [10]. *Pistacia atlantica* var. *mutica*, which is called *baneh* in local areas of Iran, is a wild pistachio (WP) from Anacardiaceae family and grows in the western, central, and eastern parts of the country. It is composed of a kernel, hull, and wooden shell [11-13]. Different parts of this plant have been used traditionally. Local residents use the fruit of the plant as a nut and its resin as a gum, mouth freshener, and treatment for gastrointestinal ulcers and skin burn wound [14-16]. Furthermore, a study showed that the antimicrobial activity of its leaves is due to its high content of phenolic compounds [17]. On the basis of previous studies, oil of WP fruit is considered a major source of oleic acid, the most abundant MUFA, and some valuable lipid-soluble bioactive compounds such as tocopherols, phenols, and phytosterols [13, 18].Therefore, the aim of this study was to investigate the preventive effects of different WP oils on oxidative stress status, liver enzymes, and histopathological findings in metabolic syndrome models.

## Materials and Methods

### Oil Preparation

Fresh fruits of WP were collected from Nurabad (Zagros forest), Fars province, Iran, in October 2016. The confirmation of the species with voucher specimen (no. 2817) was approved by an expert in the Traditional Pharmacy Department, Shiraz University of Medical Sciences, Shiraz, Iran. Different parts of the fruit (the green hull and the kernel) were separated and after drying in the shade, oil extraction was conducted using the cold press method (cold press device: calibre35 mm, Iran). The oils were filtered and centrifuged at 4000 revolutions per min for 15 min, and then, we stored the hull, kernel, and mixture oils (40 percent kernel and 60 percent hull oils) with minimum exposure to oxygen in dark bottles in the refrigerator. According to a study [19], the effective dose of olive oil was 1.3 and 1.7 mL/kg, and as the MUFA content of olive oil is about 64% and the MUFA content of WP is about 52%, the dosage used in this study was 2.2 mL/kg/day, with a coefficient of 1.27.

### Chemical Profile of the Oils

The sterol content and fatty acid composition were analyzed by a gas chromatograph machine, using a Beifen system (3420A, China). The National Iranian Standard Nos. ISIRI 9670 and ISIRI 6081 were used to calculate the sterol content. The amount of total phenols was measured using the Folin-Ciocalteu reagent at 725 nm. Results were expressed as mg of gallic acid per g of oil. The reversed-phase high-performance liquid chromatography column VIT F (Knauer Smart Line, Germany) and UV detection at 295 nm were used for the structural elucidation of tocopherol. The total tocopherol content was estimated by comparing it with standards purchased from Sigma. In addition, the International Dairy Federation spectrophotometric method was used for peroxide value determination.

### Animals

In this study, we used 72 male Sprague-Dawley rats having a mean age of 6 to 8 weeks and weight of 170 to 220 g each, purchased from the center of experimental and comparative medicine, Shiraz University of Medical Sciences, Shiraz. Animals were housed in standard conditions (temperature: 22±2^.c^, humidity: %50±5 and lighting: 12 hours light/dark cycles). They had access to ad libitum drinking water and were fed a chow diet (Pars Dam Co., Tehran, IRAN). Our study protocol was approved by the ethics committee of Shiraz University of Medical Sciences (registration number: 95-01-84-12937) and performed in accordance with the ethical standards laid down in the 1964 Declaration of Helsinki and its later amendments.

### Induction of Metabolic Syndrome

We induced metabolic syndromein the rats by administering 8 g/kg body weight per day of fructose (Merch Co., Germany) by gavaging fructose solution (as 50/50 with water) [20].

### Experimental Design

The experimental groups were selected as follows:

- Group 1, the control group (receiving 2 cc normal saline as a placebo for fructose solution and 0.5 cc normal saline as a placebo for oils per day for 10 weeks)
- Group 2, fructose group (receiving 2 cc fructose solution and 0.5 cc normal saline for 10 weeks).
- Group 3, sunflower oil and fructose group (receiving 2 cc fructose solution and 0.5 cc sunflower oil per day for 10 weeks).
- Group 4, WP mixture oil and fructose group (receiving 2 cc fructose solution and 0.5 cc WP mixture oil per day for 10 weeks).
- Group 5, WP hull oil and fructose group (receiving 2 cc fructose solution and 0.5 cc WP hull oil per day for 10 weeks).
- Group 6, WP kernel oil and fructose group (receiving 2 cc fructose solution and 0.5 cc WP kernel oil per day for 10 weeks).

### Histopathological Examination

At the end of week 10 after killing the rats, their abdomens were opened and the liver was removed for histopathological examination. Liver tissues were fixed in 10% formalin. By using microtome, 5 mm sections of the liver tissue were taken. All the slides were examined by a single-blinded pathologist under a light microscope. The pathologist noted and graded the central venous congestion, congestion and dilation of the hepatic sinusoids, and inflammation of the portal tracts (0: no change, 1: slight change, 2: moderate change, 3: severe change).

### Determination of Biochemical Parameters

At the end of study, rats were euthanized using 5 mg/kg diazepam plus 50 mg/kg ketamine through intraperitoneally administration and 5 mL of blood sample was collected by cardiac puncture from each rat. The blood samples were centrifuged and then stored at −80°C before biochemical assessments. The fasting blood sugar (FBS) was assayed by enzymatic kits (Pars Azmoon., Tehran,IRAN). Insulin concentration of serum was measured using enzyme-linked immunosorbent assay kits (Monobind Inc, USA) and IR was assayed by homeostatic model assessment (HOMA) according to the following equation: fasting glucose (mmol/L) × fasting insulin (µIU/mL)/22.5. The concentration of serumic malondialdehyde (MDA) was determined by measuring thiobarbituric acid–reactive substances using a spectrophotometric assay. In addition, superoxide dismutase (SOD) activity measured by the Zellbio kit, Germany. For measuring liver enzymes (alanine aminotransferase [ALT] and aspartate aminotransferase [AST]) by a photometric method, a bioassay analyzer was used.

### Statistical Analysis

The results were statistically analyzed in different experimental groups by SPSS software, version 24 (SPSS Inc., Chicago IL). One-way analysis of variance test (and Tukey test as post hoc) was used for normally distributed data, and Kruskal-Wallis test (and Mann-Whitney *U* test as post analysis) was used for the skewed data. Statistical significance was considered at P-value <0.05.

## Results

### Chemical Analysis of the Oils

Table-1 shows that oleic acid is the main fatty acid of WP kernel and hull oils (51.17% and 54.33%), but sunflower oil has 25% oleic acid content. On the basis of our results, the most abundant phytosterol of WP kernel and hull oils was b-sitosterol (84% and 82.44%), whereas the b-sitosterol content of sunflower oil was 61%. In our study, WP hull oil had the highest amount of total tocopherol and phenol content (1070.49 and 89.5 mg/kg) compared with WP kernel oil (350.15 and 4.25 mg/kg) and sunflower oil (580 and 1.2 mg/kg). In addition, WP hull oil had the highest peroxide value (4.2 mequiv O_2_/kg oil), whereas the peroxide value of WP kernel oil and sunflower oil was 0.8 and 0.7 mequiv O_2_/kg oil, respectively.

### Glycemic Indices

As shown in Table-2, a significant difference between FBS, insulin, and HOMA-IR was seen in the 6 groups of the study (F_ (5, 58)_= 12.172; P < 0.001 for FBS, F_(5, 58)_= 7.440; P < 0.001 for insulin and F_(5, 58)_= 2.487; P= 0.042 for HOMA-IR). There was a significant increase in FBS concentration and HOMA-IR in the fructose group compared with the control group (P< 0.05). The study showed a significant decrease in FBS and HOMA-IR in the mixture and kernel oils groups compared with the fructose group. Consumption of kernel oil also increased the insulin level significantly(P<0.05). Concentrations of FBS and insulin in the groups that consumed mixture and kernel oils were significantly lower and higher, respectively, compared with the group that consumed sunflower oil (P<0.001).

### Liver Enzymes and Oxidative Stress Parameters

Statistical tests showed no significant changes in the ALT and AST concentration among the groups at all (F_5, 58=_ 1.073; P = 0.385 for ALT and P = 0.11 for AST); however, there was a reduction in ALT concentration of all groups compared with the control group. Serum SOD and MDA concentrations were not significant among the 6 groups (F_5, 58_= 1.885; P = 0.111 for SOD and P = 0.86 for MDA, Table-2).

### Histopathological Findings

**Figure 1:**
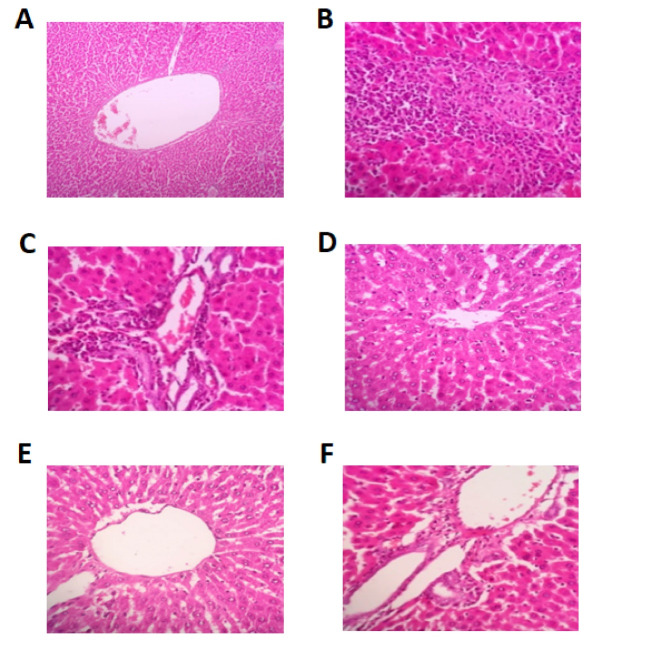
Histopathologic results of rat liver in the 6 groups. **A:** Histology of normal rat liver in control group. **B** and **C:** Histology of rat liver in fructose and sunflower oil groups with vascular congestion and lymphocytic inflammation. **D**, **E** and **F:** Histology of normal rat liver in WP mixture, hull and kernel oil groups (original magnification ×40)

Table-3 and figure-1 show that there were no significant changes in histopathology findings among the groups based on total scores of the liver tissue, which was a combination of central venous congestion, congestion and dilation of the hepatic sinusoids, and inflammation of the portal tracts scores.

## Discussion

Results of our study showed that consumption of kernel and mixture oils of WP can improve FBS and HOMA-IR index, whereas there was no change in the liver enzymes, oxidation stress indices, and liver histopathology in the rats under induction of metabolic syndrome.

Our results showed that HOMA-IR index and FBS were decreased in the kernel and mixture oils groups, and the level of insulin was increased only in the kernel oil group. Chemical analysis of WP oil showed that it was a major source of oleic acid, based on documents; consumption of diets rich in oleic acid has beneficial effects on metabolic conditions [12, 21]. Hyperglycemia can impair beta-cell turnover and secretory function of the pancreas; hence, providing dietary interventions could be helpful [21]. Previous studies showed that diets with a high content of MUFA can improve the glycemic and lipid profiles; on the other hand, it can increase the secretion of glucagon-like peptide-1, which has a therapeutic effect on IR and impaired glucose tolerance [22-24]. Ryan *et al*. showed that diets rich in oleic acid can improve insulin sensitivity, glucose transport, and endothelium vasodilatation by peroxisome proliferator–activated receptors γ activation [25]. Overproduction of reactive oxygen species may be attributed to progression of diabetes, IR, and its various complications; so improving intracellular antioxidant defense might be the best therapeutic approach [26, 27]. As WP oil has a high content of antioxidants, its hypoglycemic and insulin improvement secretion effects can be another beneficial mechanism associated with its use [12, 13]. As Coppey *et al.* showed in their study, antioxidants therapy could prevent the production of free radicals and decrease the vascular and neural trauma in induced diabetic rats [28]. Another study by Vessby *et al*. on healthy men and women showed that a high proportion of MUFA in the diet, unlike saturated fatty acids, can improve the insulin sensitivity [29]. Oxidative stress is likely to play a key role in liver dysfunction through its ability to activate stress-sensitive signaling pathways, increased levels of glucose, and possibly free fatty acids [30]. The present insignificant impact of WP oils on oxidative stress indices suggests how this intervention could not affect the liver enzymes and liver histology features. In previous studies, the WP extract has been able to improve the liver-related indices. All of the previous studies were associated with the improvement of oxidative stress, which was insignificant in this study [31, 32]. Karizno *et al.* reported that WP extract in diabetic rats can decrease protein carbonyl as an oxidative stress marker. Furthermore, in a study by Tolooei *et al*, WP extract normalized catalase activity, SOD, and MDA levels in rats with hepatic toxicity by free radical scavenging activity [32]. In addition, a study by Djerrou *et al.* showed an improvement in liver enzyme levels and antiglycogenesis activity in rabbits receiving WP oil via the rectal route [33]. Although our chemical analysis of the oils showed a high content of antioxidants, we did not notice any beneficial effects on oxidative stress and hepatic biomarkers. As the fructose solution did not make significant changes in the liver enzymes and histology compared with the control group, perhaps more time was needed to view these effects. On the other hand, it can possibly be due to our study model, which was preventive. In this study, it was observed that kernel oil has more beneficial effects than mixture and hull oils on glycemic indices, which can be attributed to their peroxide value. On the basis of the study by Farhoosh *et al.* and our measurements, WP hull oil has a higher peroxide value, that is, it might be oxidized before oil extraction due to humidity, sunlight, and oxygen even though it has high antioxidant content; hence, it does not show enough notable therapeutic effects [13]. On the basis of the results of this study, consumption of WP oil could not improve oxidative stress biomarkers in 10 weeks. Therefore, long-term preventive or treatment studies may show antioxidant effects of the oils, and it is recommended to be investigated in future studies. It is proposed that WP oils can be used in human studies in the future as well. As WP kernel oil is a rich source of oleic acid and other bioactive components, has a wide distribution in Iran, and due to its therapeutic effect shown in this study, it can be introduced as an oil source in the future. Lack of blood pressure and waist circumference measurements for the rats were some limitations of this study.

## Conclusion

In summary, this study showed that in rats under metabolic syndrome induction, kernel oil of WP decreased hyperglycemia and HOMA-IR index and improved insulin secretion. However, there was no significant change in the liver enzymes and histopathologic results between groups.

## Acknowledgment

This study was extracted from a Master of sciences thesis written by Sanaz Jamshidi and financially supported (Grant no #95-01-84-12937) by the Vice Chancellery of Research and Technology in Shiraz University of Medical Sciences, Shiraz, I.R. Iran. The authors would like to thank the Center for Development of Clinical Research of Nemazee Hospital and Dr. Nasrin Shokrpour for editorial assistance.

## Conflict of Interest

None.

**Table 1:** Chemical Composition of WP Kernel Oil, WP Hull Oil and Sunflower Oil

**Parameters**	**WP kernel oil**	**WP hull oil**	**Sunflower oil**
**Fatty acids (percent)**	16:0	10.03	23.74	7.39
	18:1	51.17	54.33	25
	18:2	32.85	5.84	63.2
	Others	5.32	15.39	4.21
**Sterols**	B-sitosterol (percent)	84	82.44	61.11
	Total (mg/kg)	3811.12	1569.47	2700
**Total tocopherol (mg/kg)**	350.15	1070.49	582
**Total phenol (mg/kg)**	4.25	89.5	1.2
**Peroxide value (mequiv O2/kg oil)**	0.8	4.2	0.7

**Table 2:** Glycemic Indices, Liver Enzymes and Oxidative Stress Parameters of Each Group Fed with Different Oils

**Variables**	**Control (mean±SD)**	**fructose** **(mean±SD)**	**Sunflower oil** **+fructose** **(mean±SD)**	**WP mixture oil** **+fructose** **(mean±SD)**	**WP hull oil** **+fructose** **(mean±SD)**	**WP kernel oil** **+fructose** **(mean±SD)**	**P-value**
**FBS** **(mg/dl)**	114.45±28.42	178.00±17.32^a^	174.72±38.38^a^	120.45±14.09^bc^	150.30±27.60^a^	123.45±24.88^bc^	<.001
**Insulin** **(µIU/ml)**	1.90±0.24	1.59±0.17^a^	1.47±0.18^a^	1.78±0.18^c^	1.63±0.22^a^	1.85±0.18^bc^	<.001
**HOMA-IR**	0.53±0.13	0.7±0.12^a^	0.64±0.19	0.53±0.10^b^	0.60±0.13	0.56±0.11^b^	0.04
**AST** ^**^ **(U/L)**	13(2-107)	2.5(2-79.25)	125(75-146)	2(2-30)	36(2.75-119.25)	65(6-122)	0.11
**ALT (U/L)**	71.18±14.98	63.70±9.38	64.27±13.40	67.54±12.17	59.20±18.65	60.36±14.50	0.38
**MDA** ^**^ **(pg/ml)**	3.92(3.58-5.25)	4.27(3.75-5.78)	3.83(3.7-4.92)	3.92(3.87-4.85)	4.00(3.76-4.20)	4.08(3.50-4.37)	0.86
**SOD** **(u/ml)**	30.30±1.49	31.82±1.04	32.24±2.81	32.45±1.19	32.29±1.62	31.65±2.44	.11

*P<0.05 considered significant ^a^ significant difference compared to the control group ^B^ significant difference compared to the fructose group ^C^significant difference compared to the sunflower oil group ^D^ significant difference compared to the kernel oil group ** non-parametric data express as median (IQR) (25^th^ percentile and 75^th^ percentile) **FBS:** Fasting blood sugar; **HOMA-IR:** Homeostatic model assessment of insulin resistance; **AST:** Aspartate aminotransferase; **ALT:** Alanine transaminase; **MDA:** Malondialdehyde; **SOD:** Superoxide dismutase

**Table 3:** Total Score of Histopathological Findings Based on Central Venous Congestion, Congestion and Dilation of the Hepatic Sinusoids and Inflammation of the Portal Tracts

	**Control** **(mean±SE)**	**Fructose** **(mean±SE)**	**Sunflower oil** **(mean±SE)**	**Mixture oil** **(mean±SE)**	**Hull oil** **(mean±SE)**	**Kernel oil** **(mean±SE)**	**P-value**
Total score of histopathological findings*	0.09±0.09	0.8±0.44	1.00±0.26	0.45±0.15	0.3±0.15	0.18±0.12	0.06

*Mean ± Standard error
